# WISH: wavefront imaging sensor with high resolution

**DOI:** 10.1038/s41377-019-0154-x

**Published:** 2019-05-01

**Authors:** Yicheng Wu, Manoj Kumar Sharma, Ashok Veeraraghavan

**Affiliations:** 1 Department of Electrical and Computer Engineering, Rice University, Houston, TX USA; 2 Applied Physics Program, Rice University, Houston, TX USA

**Keywords:** Optical sensors, Imaging and sensing

## Abstract

Wavefront sensing is the simultaneous measurement of the amplitude and phase of an incoming optical field. Traditional wavefront sensors such as Shack-Hartmann wavefront sensor (SHWFS) suffer from a fundamental tradeoff between spatial resolution and phase estimation and consequently can only achieve a resolution of a few thousand pixels. To break this tradeoff, we present a novel computational-imaging-based technique, namely, the Wavefront Imaging Sensor with High resolution (WISH). We replace the microlens array in SHWFS with a spatial light modulator (SLM) and use a computational phase-retrieval algorithm to recover the incident wavefront. This wavefront sensor can measure highly varying optical fields at more than 10-megapixel resolution with the fine phase estimation. To the best of our knowledge, this resolution is an order of magnitude higher than the current noninterferometric wavefront sensors. To demonstrate the capability of WISH, we present three applications, which cover a wide range of spatial scales. First, we produce the diffraction-limited reconstruction for long-distance imaging by combining WISH with a large-aperture, low-quality Fresnel lens. Second, we show the recovery of high-resolution images of objects that are obscured by scattering. Third, we show that WISH can be used as a microscope without an objective lens. Our study suggests that the designing principle of WISH, which combines optical modulators and computational algorithms to sense high-resolution optical fields, enables improved capabilities in many existing applications while revealing entirely new, hitherto unexplored application areas.

## Wavefront sensing: megapixel resolution

Wavefront sensing with greatly improved spatial resolution is now possible thanks to a new approach. The scheme, invented by Yicheng Wu and coworkers at Rice University in the US uses a spatial light modulator (SLM), a CMOS image sensor and a computational phase-retrieval algorithm. The optical field to be analyzed is modulated by a series of random phase patterns generated by the SLM and a series of corresponding spatial intensity measurements are made on the CMOS sensor. The data is then processed by a phase-retrieval algorithm in order to generate the phase and amplitude information of the wavefront incident on the SLM. Importantly, the team says that the scheme can provide wavefront reconstruction with a ~10-megapixel resolution, several orders of magnitude better than commercial Shack-Hartmann sensors.

## Introduction

Light behaves as a wave, which can be characterized by its amplitude and phase. However, the current imaging sensors such as complementary metal oxide semiconductor (CMOS) sensors completely lose the phase information and limit the design of conventional imaging systems to mapping all information to only the amplitude of the incoming field. This mapping is not always feasible and results in many limitations.

In contrast, the goal of wavefront sensing is to simultaneously measure the amplitude and phase of an incoming optical field. The combination of these two pieces of information enables the retrieval of the optical field at any plane, which provides a larger latitude and more flexibility in the design of imaging systems. The importance of this technique has been demonstrated in microscopy^1–3^, far-field imaging^4–6^, imaging through scattering media^7–9^, and characterization of optical components^10–12^.

Traditional wavefront sensors fall into two groups. The first group is based on geometrical optics^13–15^. Shack-Hartmann wavefront sensor (SHWFS)^13^ is the most frequently used geometric design, which builds an array of lenses in front of a CMOS sensor. Each lens provides measurements of the average phase slope (over the lensed area) based on the location of the focal spot on the sensor. To achieve high phase accuracy, many pixels are required per lens to precisely localize the spot. Thus, although the CMOS sensor has millions of pixels, the spatial resolution of the measured complex field is very low. Currently, commercial SHWFSs offer up to 73 × 45 measurement points^16^, which is useful to estimate only smooth phase profiles such as air turbulence. The second group is designed based on diffractive optics^17,18^. The phase information is encoded into interferometric fringes by introducing a reference beam. However, these interferometric systems have the following two limitations: (a) the systems are bulky and heavy due to the increased optical complexity, and (b) the systems are highly sensitive to micrometer-scale vibrations.

Can we overcome these limitations and design a noninterferometric, high-resolution (multimegapixel) system? Our key insight is to capitalize upon the field of computational imaging, which provides an elegant framework to codesign advanced computational algorithms and optics to develop new solutions to traditional imaging techniques. Many other limits that were considered fundamental have been overcome by this joint design approach. For example, superresolution microscopes such as PALM^19^ and STORM^20^ achieve subdiffraction-limit imaging by combining photoswitchable fluorophores with high-accuracy localization algorithms. Fourier ptychography^21^ offers a high space-bandwidth product using an LED array microscope with phase-retrieval algorithms. Non-line-of-sight imaging^22^ enables one to look around the corner by utilizing the time-of-flight setups and 3D reconstruction algorithms.

We recognize that the traditional wavefront sensors suffer from low spatial resolution and/or high vibration-sensitivity to directly measure the phase. Our proposed approach avoids these drawbacks by combining optical modulation and computational optimization. Specifically, we use two cutting-edge technologies. First, the current high-performance CMOS technology^23,24^ enables the production of high-resolution, high-frame-rate image sensors and spatial light modulators (SLMs). Second, recent advances in the phase-retrieval algorithms^25–29^ and computational power enable us to efficiently solve large-scale optimization problems. By combining these two technological advances, we can record high-resolution intensity measurements and indirectly recover the phase using the phase-retrieval algorithms.

Our approach is inspired by recent efforts of various research groups^30–32^ to measure the wavefront computationally using sequential captures with an SLM. However, the current techniques suffer from two limitations that we aim to directly address. First, the spatial resolution of the acquired wavefronts is limited. Second, these systems are not optimized for acquisition speed, which makes the sensor incapable of imaging dynamic scenes. On the other hand, while existing single-shot wavefront sensors achieve high frame-rate recording^33–35^, they typically rely on assumptions such as the sparsity and severely limit the applicability of these systems to generic applications.

**Fig. 1:**
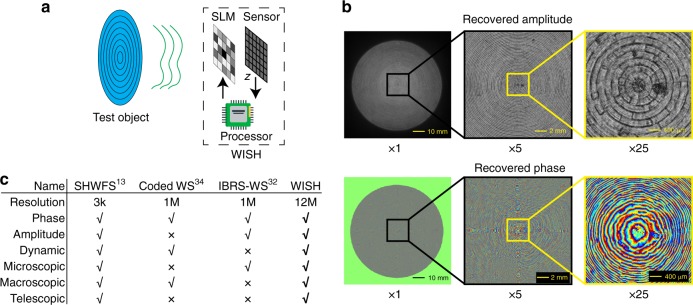
Overview of WISH. **a** WISH consists of a phase SLM, a CMOS sensor and a processor, which measures the wavefront falling on the SLM plane. The complex field of the test object (e.g., a Fresnel lens) is numerically recovered by backward propagation. **b** Reconstruction of a 76.2-mm-diameter Fresnel lens: both recovered amplitude and phase contain micron-resolution details. Details are shown in Fig. 3. **c** Comparison of wavefront sensors: we list the essential factors for SHWFS^13^ using a lenslet array, Coded WS^34^ using a binary mask, IBRS-WS^32^ using coded-diffraction-patterns, and the proposed WISH. WISH can recover an arbitrary complex field with the best spatial resolution at high frame rate and has applications in a wide range of spatial scales

In this paper, we introduce a wavefront imaging sensor with high resolution (WISH), which offers multimegapixel resolution, high frame rate, and robustness to vibrations (Fig. 1). WISH consists of an SLM, a CMOS sensor and a processor. WISH imaging works by first modulating the optical field with multiple random SLM patterns and capturing the corresponding intensity-only measurements using a CMOS sensor. Then, the acquired data are processed using a computational phase-retrieval algorithm, which estimates the complex optical field incident on the SLM. The spatial resolution of the recovered field is larger than 10 megapixels. In comparison with the traditional SHFWS, this is more than 1000 × improvement in spatial resolution. Compared with other recent designs of wavefront sensors^30–32,34,36^, WISH achieves more than 10 × improvement in spatial resolution. Although multiple shots are necessary to recover one complex field, WISH can record dynamic scenes with a frame rate of up to 10 Hz. Last but not least, because the design is reference-free, WISH is robust to environmental noise and motion, which broadens the variety of application domains where this technology can be integrated.

## Results

**Fig. 2:**
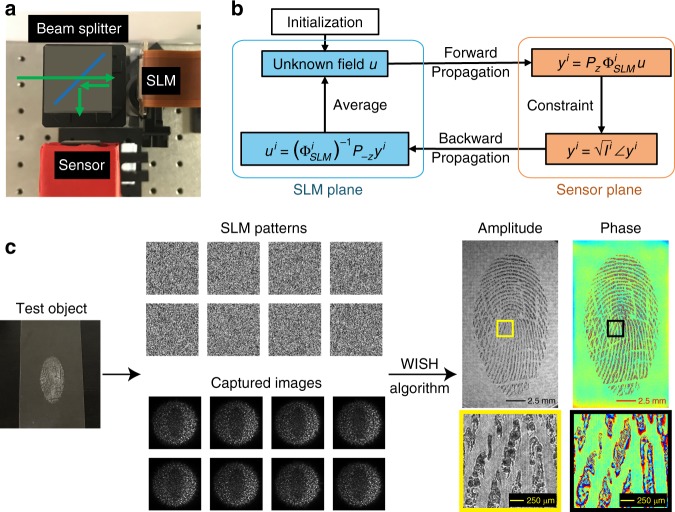
Recovering high-resolution wavefront using WISH. **a** The experimental setup of WISH. The green arrow indicates how the field propagates inside the sensor. **b** The high-resolution estimate of field *u* is recovered by iteratively enforcing the intensity measurement constraints on the sensor plane and averaging estimates from multiple patterns on the SLM plane. **c** To recover the incident wavefront from a dusted fingerprint, we projected eight random phase patterns on the SLM and captured the corresponding images. After the WISH algorithm has been performed, the high-resolution amplitude and phase reconstructions are recovered

To validate the proposed wavefront sensing technique, we constructed a table-top prototype (Fig. 2a). We illuminated the system with green light generated using a 532-nm-wavelength module diode laser (Z-LASER Z40M18B-F-532-PZ). We modulated the phase distribution of the incident light using a phase-only SLM (HOLOEYE LETO, 1920 × 1080 resolution, 6.4 µm pitch size). Because the SLM operates in the reflective mode, we inserted a 25.4-mm beam splitter to guide the field into the sensor. The distance between the SLM and the sensor is ~25 mm. The sensor is a 10-bit German Basler Ace camera (acA4024-29um) equipped with a Sony IMX-226 CMOS sensor (1.85 µm pixel pitch, 4024 × 3036 resolution).

During the acquisition, multiple phase modulation patterns were projected onto the SLM. The SLM patterns modulated the incoming optical field before propagating towards the sensor, which recorded 2D images that corresponded to the intensity of the field at the sensor plane. The phase information of the modulated field was not recorded^37^. Multiple uncorrelated measurements were recorded with different SLM patterns to enable the algorithm to retrieve the phase. In an ideal setting, the SLM pattern should be fully random to diffract the light to all pixels of the sensor to improve the convergence and accuracy of the iterative retrieval algorithm^30,36^. However, the cross-talk effect from the SLM becomes a serious issue, especially for high-frequency patterns, which deteriorates the quality of the recovered image^30,31^. Moreover, due to the finite size of the sensor, the SLM should only diffract light to the level that the sensor can capture most of the signal. In our experiment, the SLM patterns are first generated by low-resolution random matrices and subsequently interpolated to match the SLM resolution (see the Methods section for more details on SLM pattern designing).

Mathematically, for each measurement *I*^*i*^ captured with the corresponding random phase modulation ${\Phi^i_{{SLM}}}$, the forward model is as follows:

1 $$\sqrt {I^i} = \left| {P_z(\Phi _{{{SLM}}}^i \circ u)} \right|$$

where *u* is the unknown field that falls on the SLM. The symbol “∘” denotes Hadamard product to represent the elementwise multiplication between the phase on the SLM and the field. *P*_z_ is the propagation operator (at the propagating distance *z*), which is modeled as Fresnel propagation^37^ (see Methods).

To estimate field *u* from *K* measurements, we form the following optimization problem:

2 $$\hat {u} = \arg \min_{u}\mathop {\sum}\nolimits_{i = 1}^{K} \left\| {\sqrt{I^{i}} - \left| {P_{z}\left( {{\Phi }_{SLM}^{i} \circ u} \right)} \right|} \right\|$$

This is a phase-retrieval problem, which is nonlinear and nonconvex. There are many quality algorithms to solve such problem^25–27^. Here, we apply Gerchberg-Saxton (GS) algorithm^25^ to recover the field *u* by alternating projections between the SLM and the sensor plane, as illustrated in Fig. 2b. The detailed derivation and implementation of the algorithm can be found in Supplemental Section S1.

To correctly recover the unknown field, a minimum number of measurements *K* is required for the algorithm to converge. Intuitively, a more complicated field *u* requires more measurements as an input. When the prior information of the unknown object is available, such as the sparsity or support, potentially far fewer measurements are required^33,35,38^. In our work, no constraint is applied to the unknown field to make our sensor valid for objects with an arbitrary phase distribution. More discussion can be found in Supplemental Section S2, and the number of measurements for each experiment is listed in the Method Section.

The resolution of WISH is determined by the pixel size of the SLM *δ*_*SLM*_, pixel size of the camera sensor *δ*_*sensor*_, and distance *z* between them. As shown in the supplementary information (Section S3), in most cases when *δ*_*SLM*_ is larger than *δ*_*sensor*_, the resolution is limited by *δ*_*sensor*_, as long as *z* is sufficiently large to enable each sensor pixel to receive the field from multiple SLM pixels. As a result, although smooth SLM patterns (i.e., large effective SLM pixel size) are used in our experiment, WISH offers the full sensor resolution.

To experimentally demonstrate how WISH works, we imaged a fingerprint on a glass microscope slide with dusting powder, which is placed ~76 mm from the sensor. As shown in Fig. 2c, eight random patterns were sequentially projected on the SLM, and the corresponding images were captured by the CMOS sensor. Based on the introduced WISH algorithm, both amplitude and phase were retrieved with high resolution. The phase distribution of the ridge patterns significantly varies because the fingerprint powder randomly scatters light.

Since WISH offers the unique capability to simultaneously measure the amplitude and phase in high resolution, it becomes a powerful tool to solve the inverse imaging problems with deterministic or even random transfer functions. Here, we introduce three applications to demonstrate that WISH can be applied to the full gamut of spatial scales. In the telescopic scale, for the first time, we demonstrate the diffraction-limited high-resolution imaging using a large-aperture but low-quality Fresnel lens. In the macroscopic scale, we show the utility of WISH for obtaining high-resolution images of objects obscured by scattering. In the microscopic scale, we convert WISH into a lensless microscope for biological imaging with high spatial and temporal resolution.

### Example application I: long-distance, diffraction-limited imaging with a Fresnel lens

**Fig. 3:**
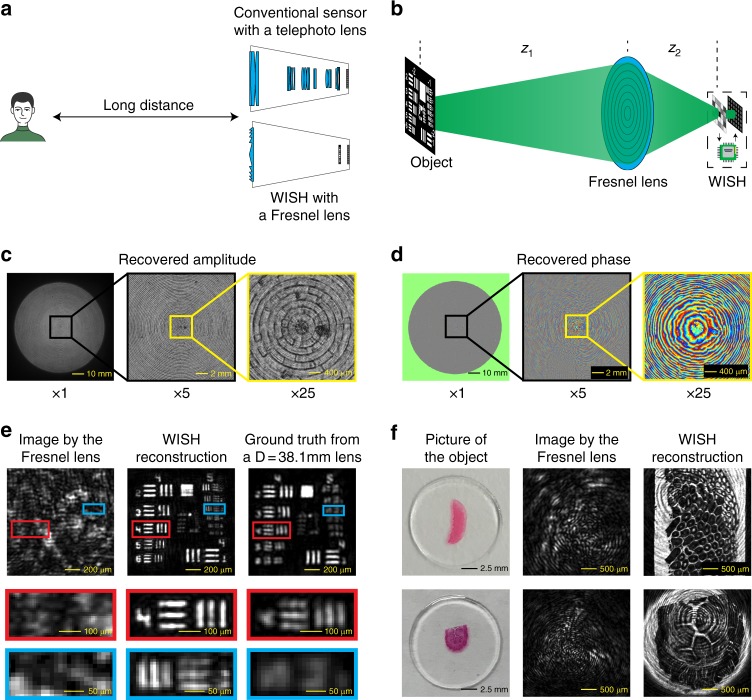
Micron-resolution imaging from meters away by combining WISH and a Fresnel lens. **a** For long-distance imaging, a conventional imaging sensor needs a telephoto lens to capture high-quality images. Using WISH, a large-aperture and low-quality Fresnel lens is sufficient to achieve the same performance. **b** Illustration of the experimental setup. **c**, **d** Recovered amplitude and phase of a 76.2-mm-diameter Fresnel lens after the calibration process, respectively. **e** Results of the USAF resolution target. Left: directly captured image with the Fresnel lens. No features are recognizable in the image. Middle: recovered intensity based on our algorithm. The best-resolved feature is a 12.40-μm line width at group 5, element 3. Right: ground truth by replacing the Fresnel lens with a high-quality lens with a 38.1-mm-diameter aperture. The best-resolved feature is a 22.10-μm line width at group 4, element 4. These results indicate that we recover almost the diffraction-limited resolution with the 76.2-mm-diameter Fresnel lens. **f** Two biological microscope slides from rabbit testis and dog esophagus are tested. Left: close-up photos captured by a cell-phone camera. Middle: directly captured image with the Fresnel lens. Right: recovered intensity based on the WISH algorithm

In many optical imaging or computer vision applications such as astronomical observation, satellite imaging, and surveillance, the imaging device is located very far from the object. However, to capture a photograph of a person 1 km away using a conventional sensor, for example, requires a telephoto lens, which contains dozens of single lenses to accommodate for diffraction blur and aberrations (Fig. 3a). Instead, we propose to combine WISH with a light and inexpensive Fresnel lens to achieve the same performance. The Fresnel lens plays the following two important roles here: (a) increasing the effective aperture size and (b) focusing light onto a small region to improve the signal-to-noise ratio. However, by itself, a Fresnel lens cannot produce a high-quality image on the sensor due to aberrations and distortions^39^. WISH enables us to computationally compensate for these aberrations and distortions, thereby achieving compact, large-aperture, diffraction-limited imaging.

To demonstrate this capability, we captured images of objects at 1.5 m away using a 76.2-mm diameter Fresnel lens (Fig. 3b). The complex object *x* is illuminated with a known phase distribution *L* (e.g., constant phase for a collimated light source or quadratic phase for a point source). The wavefront propagates distance *z*_1_ before hitting the Fresnel lens. Fresnel lens *F* gathers the field inside the entire aperture to the wavefront sensor at distance *z*_2_. The forward model is described by the following:

3 $$u_3 = P_{z_2}(F \circ P_{z_1}(L \circ x))$$

After the complex field *u*_3_ has been retrieved by WISH, object *x* can be obtained by backward propagation as follows:

4 $$x = L^{{\mathrm{ - 1}}} \circ P_{ - z_{\mathrm{1}}}{\mathrm{(}}F^{{\mathrm{ - 1}}} \circ (P_{ - z_{\mathrm{2}}}u_{\mathrm{3}}{\mathrm{))}}$$

However, *F* contains unknown aberrations and must be calibrated beforehand. During the calibration, the object is removed so that the incident light directly shines on the Fresnel lens. In this case that *x* = 1, we recover the corresponding field *u*_30_ by WISH on the SLM plane. Then, based on Eq. 3, we can calculate the lens field as follows:

5 $$F = {\mathrm{(}}P_{z_{\mathrm{1}}}L{\mathrm{)}}^{{\mathrm{ - 1}}} \circ P_{ - z_{\mathrm{2}}}u_{{\mathrm{30}}}$$

The calibration process is required only once for a given lens and does not need to be repeated as the object or setup changes. Figures 3c, d show the calibrated amplitude and phase of the Fresnel lens (Edmund Optics #43-013) with a 254-mm focal length. Zoomed-in images at ×5 and ×25 demonstrate details of the recovered field. The amplitude shows that the lens has an effective diameter of ~76.2 mm, which mainly consists of concentric circles with imperfections due to manufacturing defects. The phase is roughly quadratic with large aberrations.

For the quantitative evaluation, we used a standard 1951 USAF resolution test chart as the object (Fig. 3e). First, we directly captured images using a Fresnel lens with zero phase on the SLM, as shown in the left column. Due to the huge aberrations from the Fresnel lens, none of the features are recognizable in the image. After reconstruction, the result in the middle column shows that the features can be well resolved up to group 5, element 3 (12.40 µm line width). Because it is very difficult to find an aberration-free lens with the same aperture size as the Fresnel lens for direct comparison, we captured the ground truth images using a high-quality lens (Thorlabs AC508-250-A-ML and a 38.1-mm diameter aperture), whose diameter is half of that of the Fresnel lens. The best resolvable feature captured by the high-quality lens is group 4, element 4 (22.10 µm line width). Since the diffraction blur size is inversely proportional to the aperture size, we can infer that the smallest visible line for a diffraction-limited 76.2-mm-diameter lens is 11.05 µm wide, which is similar to the resolution in our reconstruction. Thus, WISH can nearly achieve the diffraction-limited resolution using a large and highly aberrated Fresnel lens.

In addition, we tested two prepared biological microscope slides from AmScope to demonstrate that microfeatures can be recovered at 1.5 m. Compared with the USAF target, these samples are more challenging because they are not binary, which reduces the contrast between the foreground and the background. In Fig. 3f, the first column is a cross-section of rabbit testis and shows 200-μm-diameter cells recognizable with fine details such as the nuclei and membrane. The second column is a cross-section of dog esophagus. In comparison with the entirely distorted image, which was directly captured from the Fresnel lens, our reconstructed image shows clear blood vessels that are 20–150 μm in diameter. There are ring artifacts on the background due to a small misalignment in the experiment.

### Example application II: imaging through scattering media

**Fig. 4:**
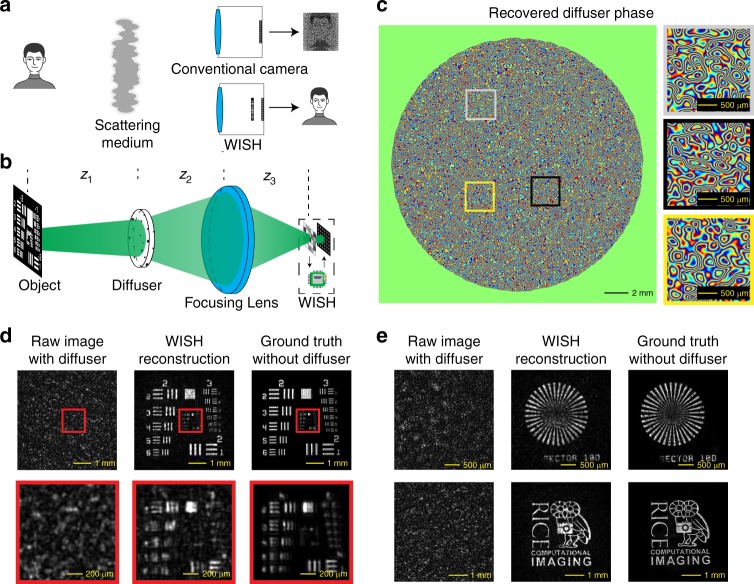
Looking through a diffuser without losing resolution by WISH. **a** To image an object (e.g., a person) through a scattering medium, conventional cameras only record random speckle patterns, while WISH can reconstruct the clear object. **b** Illustration of the experimental setup. **c** Recovered phase of the diffuser after the calibration process. The wavefront that reaches the diffuser is scattered due to these random phase aberrations. **d** The USAF resolution target is used to evaluate the resolution of our system. The left column plots the captured images with the diffuser. The middle column shows the recovered images. For comparison, the images captured without the diffuser are treated as the ground truth and shown in the right column. **e** Results for a Siemens star and our lab logo. These results show that WISH can recover a hidden object without losing the resolution

Seeing through fog or beneath the skin is an extremely challenging task due to scattering. As shown in Fig. 4a, if a person is hidden behind scattering media, most of the key features are lost when captured by a conventional camera. It has been shown that the transfer function of volumetric scattering media can be modeled as a complex linear system (called the scattering matrix or transmission matrix), and this system can be inverted (the effects of scattering are undone) if complete field measurements can be obtained at the sensor^8,28,40,41^. By measuring the phase distortion and computationally inverting it, WISH can reconstruct the objects hidden by a thin scatterer. As plotted in Fig. 4b, the wavefront from the object is scattered by a highly random diffuser *D* at distance *z*_1_. A focusing lens, which is modeled as quadratic phase distribution Φ_*lens*_, collects diffused light to WISH. However, this lens is not mandatory if the diffuser is near the sensor.

First, we calibrate the diffuser by illuminating it with collimated light from the far side. WISH measures the scattered field on the SLM plane *v*_40_, and the diffuser field can be calculated as follows:

6 $$D = P_{{\mathrm{ - }}z_{\mathrm{2}}}{\mathrm{(}}{{\Phi }}_{{{lens}}}\circ P_{{\mathrm{ - }}z_{\mathrm{3}}}v_{{\mathrm{40}}}{\mathrm{)}}$$

After the calibration, a hidden object is placed behind the diffuser. Based on the recovered field *v*_4_ from WISH, the field of the object can be recovered by numerical backward propagation as follows:

7 $$x = P_{ - z_1}(D^{ - 1} \circ P_{ - z_2}({{\Phi }}_{lens} \circ P_{ - z_3}v_4))$$

To test the system, we imaged various objects through a 25.4-mm diameter diffuser (Edmund Optics 47-988). The objects were placed 80 cm behind the diffuser and illuminated by a collimated laser beam. After light from the object passes through the diffuser, the wavefront is converged by a 50.8-mm-diameter lens with 180-mm focal length (Thorlabs AC508-180-A) and captured by WISH (*z*_3_ = 18 cm).

The calibrated phase profile of the diffuser is plotted in Fig. 4c. The left side shows the entire diffuser, which is 23.8 mm in diameter, while the right side provides three magnified regions. This phase map corresponds to the physical height of the structures on the diffuser, which randomly diffracts light and causes large phase aberrations. For the direction with the largest gradient, a 2π phase shift is ~7 pixels (31.5 μm). The amplitude part of the diffuser is almost flat and unimportant since the diffuser does not absorb much light.

We captured images of the USAF resolution chart to evaluate the performance of the reconstruction (Fig. 4d). The left column shows direct-captured images with the diffuser. Due to the random phase from the diffuser, this image contains only speckle patterns with no visible information under coherent illumination. In the middle column, the distortion from the diffuser is computationally removed, and the object is recovered using our proposed method. For comparison, the images captured without the diffuser are displayed in the right column. The center regions, which are highlighted in red, are magnified and presented in the bottom row. For the reconstruction, the best resolvable feature is group 4, element 2 with a bar width of 27.84 µm. The ground truth (without the diffuser) shows that the smallest feature size is 24.80 µm (group 4, element 3). Although the diffuser completely destroys the field, our algorithm removes nearly all distortions and recovers the object. Since there is no object constraint in our algorithm, various objects can be similarly reconstructed. Figure 4c shows the results from a Siemens star and our lab logo. Although the raw captured images look random due to the diffuser, the reconstruction is comparable to the ground truth captured without the diffuser.

Similar to Katz et al.^8^, it is straight-forward to expand our method from the transmissive mode to the reflective mode, which can be useful for applications such as looking around the corner with a diffused wall^22,42^.

### Example application III: lensless microscopy

By bringing samples near the sensor, we can convert WISH into a lensless microscopy system. Lensless imaging techniques can result in extremely lightweight and compact microscopes^43^. Pioneering work has demonstrated the applications in holography^44^, fluorescence^45^, and 3D imaging^46,47^. Although a lens-based microscope has a tradeoff between the field of view (FOV) and resolution, lensless microscopy offers high-resolution while maintaining a large FOV.

**Fig. 5:**
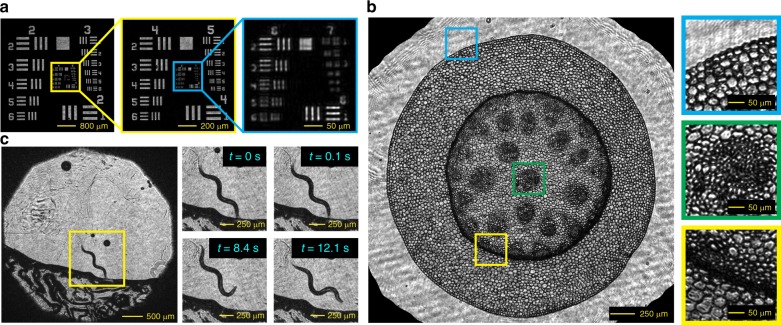
WISH for lensless microscopic imaging. **a** Reconstruction of a USAF resolution target with a large FOV (5 mm × 5 mm) and a high resolution (4.92 μm bar width). **b** The reconstruction of a cross-section of the lily-of-the-valley. Three zoomed-in images show details of the characteristic regions. **c** Four frames from a reconstructed video (10 Hz) of a Caenorhabditis elegans living on agar

We tested WISH as a lensless microscope by measuring a standard resolution target and biological samples. As shown in Fig. 5a, we imaged a large region of a USAF target with the smallest visible features in group 6, element 5 (4.92 μm bar width). Currently, due to the necessity of a beam splitter, the resolution is limited by the space between the sample and the SLM. Replacing the reflective SLM by a transmissive SLM is a potential solution to increase the spatial resolution. Figure 5b shows the reconstruction of cells from lily-of-the-valley (Convallaria majalis). Three subregions are magnified to show the characteristic features in the sample.

The ability to observe a dynamic scene is also crucial to understand the behavior of the live samples. By optimizing the syncing between the SLM and the sensor, we achieve acquisition speeds of up to 20 Hz (see Methods). During the reconstruction, eight frames are input to the algorithm with a sliding window of two frames, which results in a recovered video with a 10-Hz frame rate. Assuming that the change between neighboring frames is small, the converged reconstruction from the previous frame can be used as the initialization of the next frame, which significantly speeds up the reconstruction. As an illustration, we captured a video of a Caenorhabditis elegans living on agar. Several frames from the reconstructed video are shown in Fig. 5c. Although the current prototype can only achieve ~10 Hz high-resolution full-wavefront imaging, this is not a fundamental constraint of the proposed design but a limitation imposed by the choice of SLM. By using faster SLMs^48^, we can achieve 100–1000 Hz, high-resolution, full-wavefront sensing capabilities using the WISH design.

## Discussion

We have demonstrated a high-resolution, noninterferometric wavefront sensor, termed WISH. This computational-imaging-based method shifts the complexity from hardware to algorithm and offers the ability to measure highly variant optical fields at more than 10-megapixel resolution. Experimentally, we show that WISH can both recover objects at high resolution and perform diffraction-limited reconstruction in highly distorted optical systems. The versatility of our sensor can significantly improve the performance of existing technologies such as adaptive optics and microscopy while providing a new tool for emerging fields including imaging through scattering media and biomedical and scientific imaging.

Although WISH is a powerful and promising computational imaging technique, its current form still suffers from a few limitations that need to be addressed in the future. Currently, WISH must sequentially capture at least eight images to accurately recover a field. Two improvements are necessary for WISH to achieve the same goal using a single shot. The phase-retrieval method should be improved to reduce the number of measurements required as input, and a camera array system must be built, where each sensor captures a modulated field with a random phase mask. Moreover, although the GS-type algorithm yields accurate phase retrieval under random modulation, it takes hundreds of iterations to converge. Modern optimization techniques may be developed using advanced tools and software for machine learning to improve the runtime. Careful exploration of the space of optimization algorithms is a nontrivial task, which we are actively pursuing in the future. In addition, our current prototype is not optimized for size. A beam splitter is necessary due to the reflective-mode SLM. Instead, a transmissive-mode SLM will make the system more compact. Tunable metasurfaces^49^, which enable thin and lightweight optical elements with precisely engineered phase profiles, can also be integrated. Finally, the current algorithm can only handle static aberrations because we calibrate aberrations prior to the measurement and assume that they remain unchanged during the acquisition process. Designing an optimization framework to automatically separate the object and aberrations without calibration is of great interest to applications such as autonomous driving (in challenging weather) and imaging beneath the skin.

## Materials and methods

### SLM patterns design

The SLM pattern should satisfy three requirements. First, to improve convergence and reduce noise, the field from multiple SLM pixels should be able to randomly interfere. Second, to increase the signal-to-noise ratio, the field should not be scattered too much to ensure that the sensor collects most of the scattered light. Third, to reduce the impact of the cross-talk effect, the pattern should be locally smooth. For each SLM pattern in our experiment, we first generated a 192 × 108 random matrix with a uniform distribution of 0–1. Then, we upsampled the matrix by a factor of 10 using bicubic interpolation in MATLAB to create grayscale images of resolution 1920 × 1080. These grayscale images were used as the SLM patterns.

### Numerical propagation model

The numerical propagation is modeled as a Fresnel propagation (FP)^50^ as follows:

8 $$U\left( {r_2} \right) = P_z\left\{ {U\left( {r_1} \right)} \right\} = Q\left[ {\frac{1}{z},r_2} \right]V\left[ {\frac{1}{{\lambda z}},r_2} \right]{\cal{F}}\left[ {r_1,f_1} \right]Q\left[ {\frac{1}{z},r_1} \right]\{ U\left( {r_1} \right)\}$$

The output field *U*(*r*_2_) is computed (from right to left) by multiplying the input field by a quadratic phase (*Q*), Fourier transforming (ℱ), scaling by a constant phase (*V*) and multiplying by another quadratic phase factor (*Q*). Although the angular-spectrum propagation (ASP) is more accurate in theory, both FP and ASP gave nearly the same result in our current setup. In addition, FP has two advantages: (1) there is only one Fourier transformation (FT) instead of two in ASP, which reduces the computation in the iterative algorithm, and (2) the grid spacing in the input and output planes must be identical for ASP, while FP can have different spacings in the input and output planes. Thus, FP can save unnecessary sampling for the case when the input and output fields have notably different sizes (e.g., recovering the wavefront of a large-aperture Fresnel lens from WISH).

### Image acquisition and reconstruction

In the experiment, we used 32 patterns for the setup with the Fresnel lens (Figs. 1 and 3) and diffuser (Fig. 4) and 8 patterns for the other experiments (Figs. 2 and 5). For each SLM pattern, we acquired two 10-bit images and averaged them to reduce the effect of noise. No high-dynamic range (HDR) measurement was required.

During the reconstruction, we split the data into batches, where each batch contained four SLM patterns and their corresponding measurements. All batches were individually processed in an NVIDIA Tesla K80 GPU with 12 GB RAM and averaged in each iteration.

### Video recording for dynamic scenes

The LETO SLM provides a synchronization signal at 60 Hz, which is used to trigger the CMOS sensor. Due to the delay between sending the phase pattern and refreshing it on the SLM, we changed the SLM patterns at 20 Hz and kept only the last frame for every three frames captured from the sensor to ensure that the captured image was stable.

## Supplementary information

Final-version Supplementary information
