# Supplementary material for: WISH: wavefront imaging sensor with high resolution
Source: Light Sci Appl. 2019 May 1;8:44. doi: 10.1038/s41377-019-0154-x (PMC6491653; doi:10.1038/s41377-019-0154-x)
Supplement: Supplementary file 1 — Final-version Supplementary information [file 41377_2019_154_MOESM1_ESM.docx]

**WISH:** **Wavefront Imaging Sensor with High Resolution**

**Supplementary information**

Yicheng Wu^1,2^, Manoj Kumar Sharma^1^ and Ashok Veeraraghavan^1,2, *^

^1^*Department of Electrical and Computer Engineering, Rice University, Houston, TX, USA*

^2^*Applied Physics Program, Rice University, Houston, TX, USA*

**Corresponding author. E-mail: vashok@rice.edu*

**S1. WISH algorithm**

Without loss of generality, let’s consider the 1D case and ignore boundary effects. The forward model is

$$\sqrt{I^{i}}=\left| P_{z}\Phi_{SLM}^{i}u \right|= \left| A^{i}u \right| ( SEQ Equation \backslash* ARABIC 1)$$

For multiple SLM patterns, the measurement matrix can be stacked together.

$$\sqrt{I}=\left( \begin{matrix} \sqrt{I^{1}} \\ \vdots\\ \sqrt{I^{K}} \end{matrix} \right)=\left( \begin{matrix} \left| A^{1}u \right| \\ \vdots\\ \left| A^{K}u \right| \end{matrix} \right)=\left| Au \right| ( SEQ Equation \backslash* ARABIC 2)$$

To estimate the field$u$, the optimization becomes

$$\hat{u}=arg\min_{u} \left\| \sqrt{I}-\left| Au \right| \right\| ( SEQ Equation \backslash* ARABIC 3)$$

Since $A$ is not a square matrix, pseudo-inverse $A^{+}$ is used here

$$A^{+}=\left( A^{*}A \right)^{-1}A^{*}=\frac{1}{K}A^{*} ( SEQ Equation \backslash* ARABIC 4)$$

$A^{*}$is the conjugate transpose of $A$. Based on the definition of $A^{i}$, the conjugate transpose is

$$A^{i*}=\left( \Phi_{SLM}^{i} \right)^{-1}P_{z}^{-1}=\left( \Phi_{SLM}^{i} \right)^{-1}P_{-z} ( SEQ Equation \backslash* ARABIC 5)$$

A good property of the propagation operator is that its inverse is the backward-propagation operator.

Now, if we define the complex field on the sensor as $y^{i}=\sqrt{I^{i}}exp(j\theta^{i})$, we can write the recovered field $\hat{u}$ as follows

$$\hat{u}=A^{+}y=\frac{1}{K}\left( \begin{matrix} A^{1*} & \cdots& A^{K*} \end{matrix} \right)\left( \begin{matrix} y^{1} \\ \vdots\\ y^{K} \end{matrix} \right)=\frac{1}{K}\sum_{i=1}^{K} \left( \Phi_{SLM}^{i} \right)^{-1}P_{-z}y^{i} ( SEQ Equation \backslash* ARABIC 6)$$

This formula says that the estimated signal is the average of all different measurements backward-propagated before the SLM pattern.

The iterative algorithm works as follows (Fig. 2b in the main text). The complex field *u* is first initialized by taking the average fields propagated back from the sensor plane with captured amplitudes and zero phases. In each iteration, the field *u* modulated by different SLM patterns $\Phi_{SLM}^{i}$ and propagates distance *z* to the sensor plane. For each complex field $y^{i}$ at the sensor plane, the amplitude is replaced by the corresponding measurement $\sqrt{I^{i}}$. Next, these fields are propagated back to the SLM plane. According to the discussion above, $u^{i}$ from different measurements are averaged for the next iteration. The estimation will finally converge to the desired solution.

**S2. Required number of measurements**

To estimate the field *u* correctly, it is critical to pick the number of measurements *K* properly. Here, we show a quantitative evaluation on how *K* affects the recovered results in 2-D simulations. Specifically, the unknown field is a 64×64 random complex matrix with 1 μm pixel size. Both the SLM and sensor have 512×512 pixels with 1μm pixel size. The propagation distance between the SLM and the sensor is 500 μm and numerical propagation is calculated by the angular spectrum method. Gaussian noise with 0.01 standard variation is added to all measurements. The error is defined as follows,

$$Error= \frac{\left\| \left| \hat{u} \right|-\left| u_{GT} \right| \right\|_{2}}{\left\| u_{GT} \right\|_{2}} ( SEQ Equation \backslash* ARABIC 7)$$

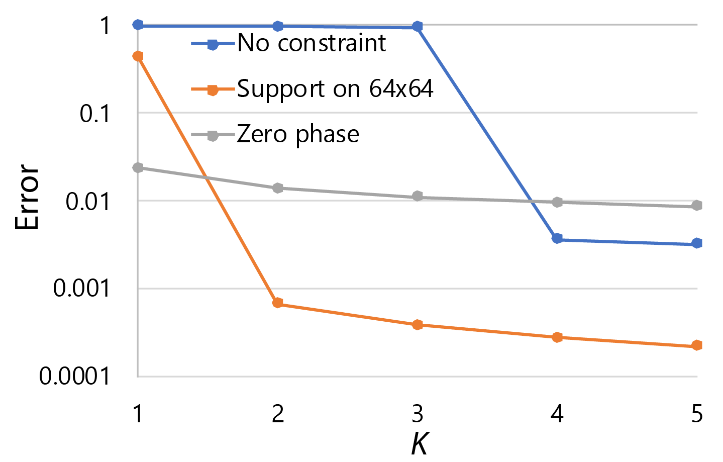


**Figure S1: Reconstruction performance for different numbers of measurements K.** There is a phase transition that the reconstruction algorithm is able to converge once the number of the measurements is large enough. Three cases correspond to no prior knowledge, knowing the field is restricted in a 64×64 region, and knowing the field only has amplitude information, respectively. Fewer measurements are required when prior information is available.

As shown in Fig. S1, when there is no constraint on the unknown field, at least four measurements are required to estimate the field correctly. There is a huge error difference from not converging to converging when *K* is larger than the threshold. Once *K* overpasses the minimum requirement, increasing the number of measurements improves the performance slightly by reducing the noise. Next, by adding support confining the field in the 64×64 region, two measurements are sufficient to recover the field. Alternatively, if we know that the unknown field only contains amplitude information with zero phase beforehand, we can find the correct estimation even with one measurement. It means the number of measurements we need is affected by the prior knowledge significantly.

**S3. Wavefront sensor resolution analysis**

In order to analysis the sensor resolution, we need to take a close look at the structure of the forward model (Eq. 1). When the propagation distance *z* is short, $P_{z}$ is a band matrix since one pixel of the incident field will fall on a local region of the sensor after its propagation. As *z* increases, the width of the band increases. When *z* is large enough that Fraunhofer approximation^1^ is satisfied, $P_{z}$ becomes a Fourier transformation. To make our setup compact, we keep *z* to be short (~30 mm). $\Phi_{SLM}$ is a diagonal matrix to have element-wise multiplication with the field $u$. The combined matrix $P_{z}\Phi_{SLM}^{i}$ is called the measurement matrix $A^{i}$, which is essentially a weighted propagation matrix where each column is multiplied by the phase from the SLM. To be able to recover an unknown pixel on $u$, we need to make sure multiple uncorrelated measurements can be applied to this pixel when different random SLM patterns are projected.

We will discuss three different scenarios about the pixel of unknown field *δ_field_*, SLM *δ_SLM_*, and sensor *δ_sensor_*, to find out the resolution limit. The measurement matrixes for different conditions are plotted in Fig. S2. The background band matrix is the propagation matrix, and the colored columns show the weighting by different SLM patterns. Each color corresponds to one SLM pixel, indicating one independent phase shift.


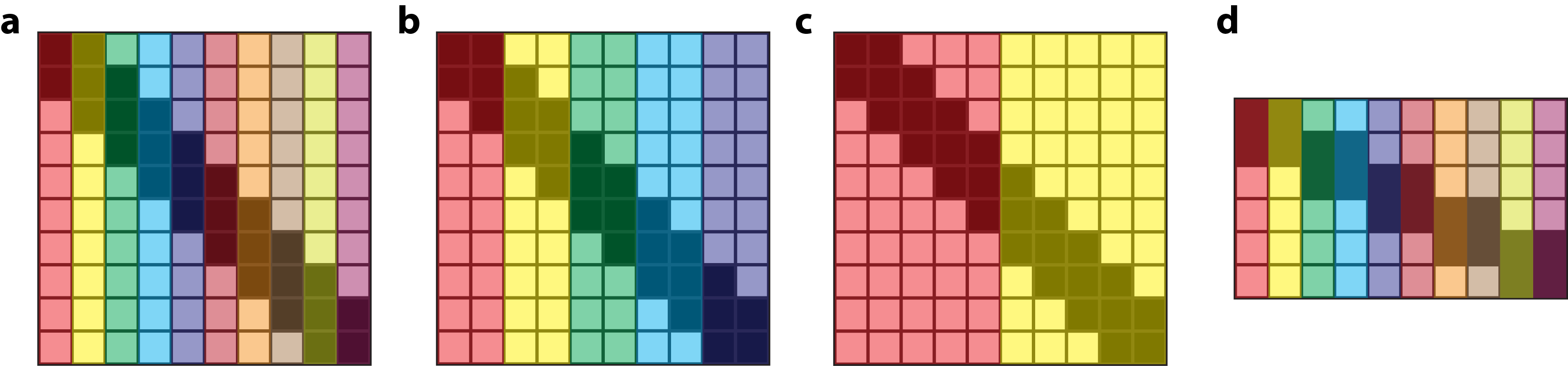


**Figure S2: Measurement matrix under different conditions. (a)** *δ_SLM_* = *δ_field_* = *δ_sensor_*, the algorithm can recover the unknown field with full resolution. **(b)** *δ_SLM_* = 2*δ_field_* = 2*δ_sensor_*, the algorithm can still recover the unknown field with full resolution. **(c)** *δ_SLM_* = 5*δ_field_* = 5*δ_sensor_*, the algorithm cannot recover the unknown field with full resolution. **(d)** *δ_sensor_* = 2*δ_field_* = 2*δ_SLM_*, , the algorithm can recover the unknown field with full resolution if the number of SLMs is double.

1. *δ_SLM_* = *δ_field_* = *δ_sensor_*

In this case, each diagonal element of $\Phi_{SLM}$ can be changed independently, which means that the weighting on the measurement matrix can also be adjusted freely (Fig. S2a). If $\Phi_{SLM}$ is random, with high probability, different rows of the measurement matrix will be orthogonal to each other^2^. Thus, every unknown field pixel can be recovered by the algorithm as long as there are sufficient patterns. For a large propagation distance *z* when the propagation operator becomes a Fourier transformation, Candes *et al.* show theoretical guarantees about its convergence^3^.

1. *δ_SLM_* = *M* *δ_field_* = *M* *δ_sensor_*

Since the SLM has a larger pixel size, each SLM pixel modulates *M* field pixels in the same way. *M* adjacent elements on the diagonal of $\Phi_{SLM}$ are same. And the measurement matrix is weighted by block. When *M* is small, each row is still modulated by more than one SLM pixel (Fig. S2b). We are able to create incoherent measurements by changing the phase shift of these pixels. Physically, it means that each sensor pixel will collect the field from multiple SLM pixels. By varying the SLM pixel value, we change how the field from different SLM pixels interference with each other, creating new measurements. But if the SLM pixel size is so large that the field falling on the sensor pixel is just from one SLM pixel (Fig. S2c), only a global phase shift is applied on the field, which will not make any difference on the sensor measurement. Under this condition, no matter how many SLM patterns are projected, we don’t have sufficient incoherent measurements to recover the field back. But if we can increase distance *z* and have a large sensor, then the requirement of the SLM pixel size can be relaxed.

1. *δ_sensor_* = *M* *δ_field_* = *M* *δ_SLM_*

When the sensor pixel size is large, it means that the recorded signal is the sum of all sub-pixel region. As for the measurement matrix, *M* rows are added together as shown in Fig. S2(d). Since the SLM pixel size is small, each column can be modulated freely. Thus, the recovered field resolution is still the same as the resolution of the SLM, with the cost of increasing the number of SLM patterns by *M* times for sufficient measurements.

Based on the discussion 1) to 3), the resolution is

$$\delta_{field}=\min\left( \delta_{sensor}, \delta_{SLM} \right) ( SEQ Equation \backslash* ARABIC 8)$$

Next, we show 2-D simulation to support our resolution analysis. In the simulation, the unknown field is a 64×64 random complex matrix with *δ_field_* = 1 μm. The entire sensor size is 512 μm×512 μm. Propagation is simulated by the angular spectrum method. Gaussian noise with 0.01 standard variation is added to all measurements. The error is defined in Eq. 7.


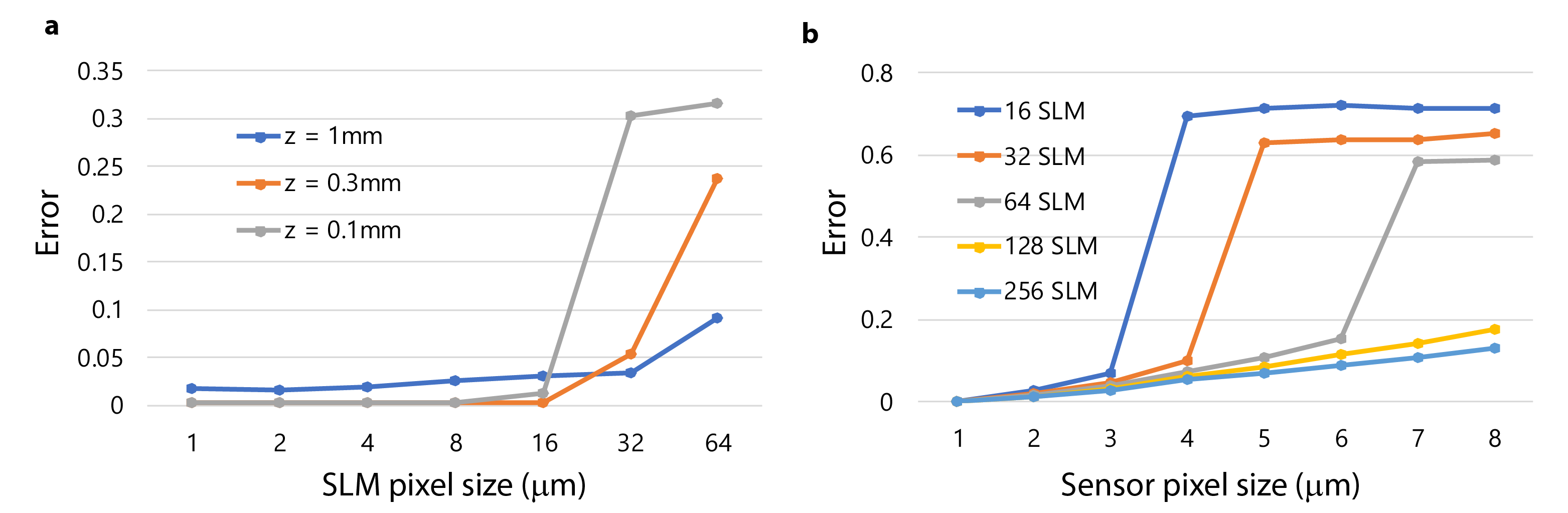


**Figure S3: Simulation results to show how the SLM pixel size and sensor pixel size affect the reconstruction error. (a)** As the size of SLM pixel increases, first the error is small and constant, then the error jumps a lot after a critical point. Different color lines correspond to different propagation distances. When SLM pixel size is small, larger propagation distance gives a higher error because more light propagates outside of the sensor. When SLM pixel size is large, long propagation distance gives a smaller error because each sensor pixel can receive light from more SLM pixels. **(b)** We fix the sensor area to be 512 μm × 512 μm. As the size of the sensor pixel increases, the number of pixels decreases, leading to a large error. But more SLM patterns can also create sufficient measurements for the algorithm to recover the correct field.

First, to show how SLM pixel size affects the reconstruction, we fix *δ_sensor_* = 1 μm and the number of SLM patterns to be 16, vary *δ_SLM_* as well as the propagation distance *z* to see the reconstruction error. As shown in Fig. S3(a), when SLM pixel size is small (i.e., *M* is small), the algorithm can recover the unknown field correctly. Then, the error jumps a lot after a critical SLM pixel size, which is decided by the condition whether the sensor pixel collects the field from one or multiple SLM pixels. This critical size increases as the propagation distance *z* increases. But large propagation distance *z* brings another practical issue because part of light propagates outside of the sensor.

Second, to show how sensor pixel size affects the reconstruction, we fix *δ_SLM_* = 1 μm and *z* = 500 μm, vary *δ_sensor_* and number of SLM patterns to see the reconstruction error. Given fixed sensor size, larger pixel size means fewer measurements, leading to large reconstruction error. To increase the number of measurements, more SLM patterns are necessary for accurate reconstruction. Results are shown in Fig. S3(b).

**S4. Sampling requirement for the Fresnel lens**

**
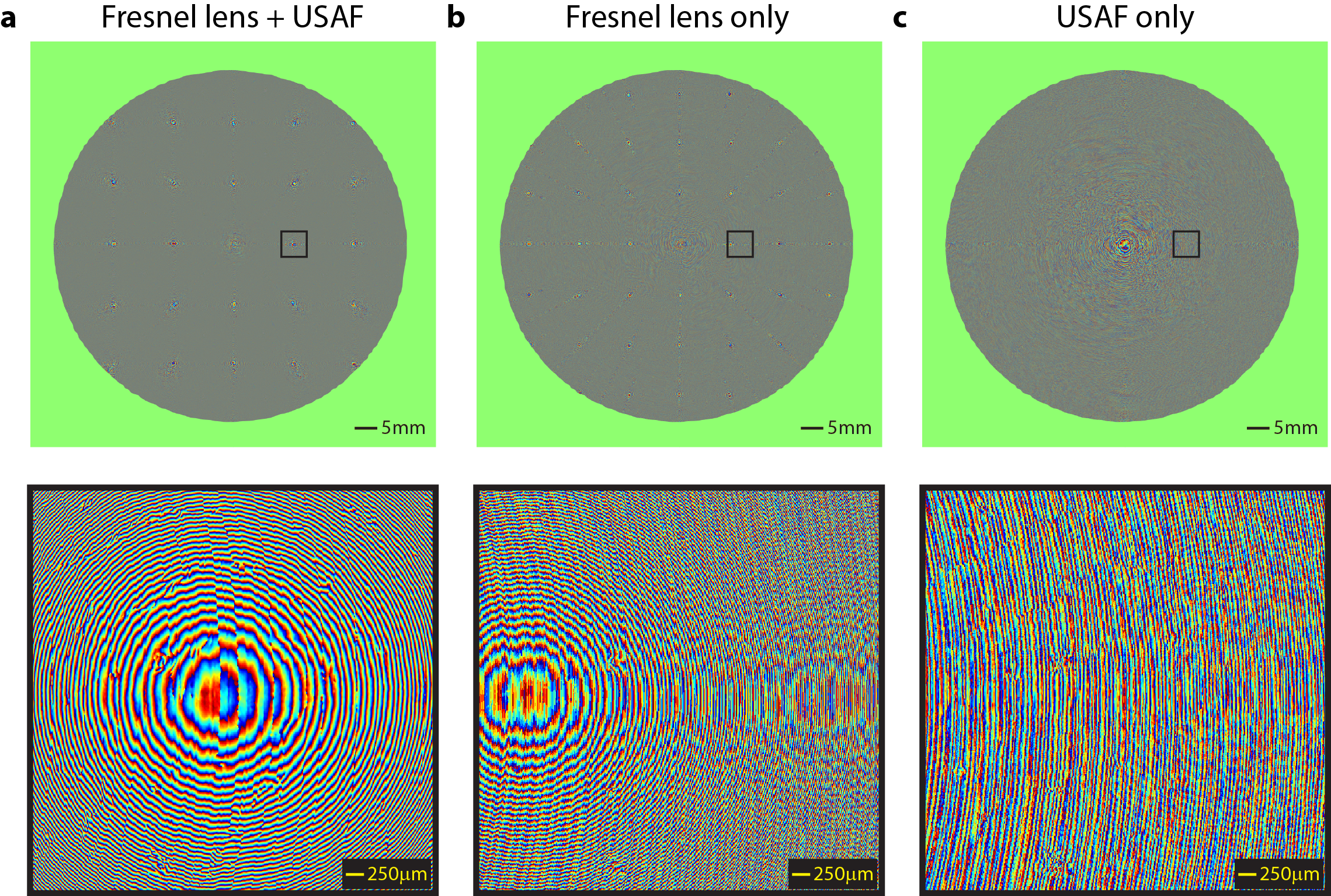
**

**Figure S4: In the long-distance imaging experiment, the sampling constraint for the Fresnel lens can be relaxed as long as the target object is not aliased. (a)** The recovered phase containing both the Fresnel lens and the USAF target. The zoom-in region shows the aliasing effect. **(b)** The calibrated phase of the Fresnel lens. It is also aliased as shown in the zoom-in figure. **(c)** The phase of the USAF itself is the difference of (a) and (b). By canceling out the high-frequency part, the left USAF phase is not aliased.

In our simulation and reconstruction, sampling requirements have to be satisfied for accurate calculation. For general wave propagation, refer to the book^4^ for detailed discussion. One special case we are dealing with is to represent the large-aperture Fresnel lens.

The phase of the Fresnel lens can be regarded as perfect quadratic phase with large aberrations. Similar to quadratic phase, the spatial frequency increases linearly as the radius increases, which means a large-size needs an extremely high sample rate. For example, supposing the Fresnel lens in our experiment (*D* = 3 inch, *f* = 10 inch) has perfect quadratic phase, we need a 40,000 × 40,000 complex matrix to represent it without aliasing artifacts. It requires over 25G memory to store such a matrix in MATLAB, and more extra memory to operate the matrix (e.g., perform an FFT). Also, our wavefront sensor only has about 10-megapixel resolution. It is impossible to measure a Fresnel lens which has 1.6 billion unknowns.

However, getting the field of the Fresnel lens *F* is not our ultimate goal. What we need is to recover the field of the unknown object falling on the Fresnel lens plane. One key insight here is that, for long-distance imaging where the distance between the object and the lens is much larger than the focal length of the lens, the object field contains much smaller spatial frequency than the Fresnel lens does on the Fresnel lens plane. Based on the Equation 4 in the main text, we remove the modulation of the Fresnel lens *F* from the entire field $P_{-z_{2}}u_{3}$ to find out the object field on the Fresnel lens plane. Even through aliasing artifacts are present in *F* and $P_{-z_{2}}u_{3}$, they are cancelled out by each other, and the remaining low-frequency object field is not aliased, as long as the sampling rate is sufficient to represent the object field. The experimental results to image a USAF resolution target with the Fresnel lens are demonstrated in Fig. S4. Section (a) corresponds to the phase of $P_{-z_{2}}u_{3}$ which is from the Fresnel lens with the USAF target. Section (b) is the phase of the Fresnel lens *F*. Both two sections contain aliasing artifacts. But the phase of the USAF target itself (Fig. S4c) is still correct. Thus, our method is not limited by the required sampling rate of the lens and save memory 36× to ensure a standard PC machine can handle the reconstruction task.

**S5. Model for scattering media**

In the section of imaging through scattering media, rather than regarding the diffuser as a transmission matrix, which blindly maps inputs to outputs, we physically model the diffuser as a thin plane with random aberrations. In this way, we dramatically shrink the number of unknowns from ${O(N}^{2})$ to$O(N)$, where $N$ is the resolution of the input field (i.e., $N$ =10^7^). As a result, the number of input fields for calibration reduces from 10^7^ to 1, making it feasible for our experiment. This thin diffuser assumption is valid for scattering media such as thin tissue and diffuse wall, indicating exciting applications in imaging beneath the skin and looking around the corner. For more complicated scattering material, we can model it as a series of 2D scattering slices between which light propagates, which has been proved to be useful for 3D reconstructions^5,6^. Combining multi-slices light-propagation model with WISH is an interesting direction for future work.

**S6. Field of view evaluation**

When the incident light rays hit WISH at a large angle, the Fresnel propagation (FP) and the phase shift of the SLM are not accurate. In our experiment, the largest FOV is about ±6°. Currently, the SLM is the main limit. As for the propagation model, as stated in the method section, although the angular-spectrum propagation (ASP) is more accurate in theory, both FP and ASP gave nearly the same result in our current setup. It means that for our current setup the Fresnel approximation is still satisfied.

Specifically, how the error from large incident angle effects three applications is evaluated below.

- Long-distance, diffraction-limited imaging with a Fresnel lens: In this case, the largest F-number we can use is 0.1 (as the FOV is about ±6°). But since our main goal is long-distance imaging, the resolution is decided by the size of the Fresnel lens instead of the F-number. By scaling up both the focal length and the size of the Fresnel lens equally, we can improve the spatial resolution of the target.
- Imaging through scattering media: Although the diffuser diffracts light in all directions, the size of the SLM limits the FOV. Thus, the portion of light measured by WISH has a small incident angle. Because of the random nature of the diffuser, we can collect signals from the object in all frequencies and get a reasonably good estimation.
- Lensless microscopy: Ideally, we want to bring the sample close to the SLM for higher resolution. In such a microscopic setting if the sample size is much smaller than the SLM, the angle of the light from the sample to a particular pixel on the SLM is fixed. We can recalibrate the phase shift of the SLM for this angle before putting into the algorithm. Combining with ASP, we should get high-resolution reconstruction. However, in the current setup, due to the beam splitter, the incident angle is small.

**S7. Reconstruction of 3D objects**

Although current results focus on 2D targets, our method may be able to achieve 3D reconstruction. There are two ways to achieve it.

First, we estimate depth based on the recovered amplitude. We back-propagate the field to multiple depths and find out the correct depth based on the gradient of the recovered amplitude (details are discussed below). As an example, three 2D bars (named as A, B, C) are located at 49.8 mm, 50 mm, and 50.2 mm. The size of each bar is 840 µm ×210 µm. Fig. S5(a) shows the amplitude of the field at 50 mm. Only bar B is in-focus, while bar A and C is out-of-focus. Although the difference is not obvious visually, we can quantitively evaluate it based on the following metric, which is the variance of gradients. For common sharp images, the intensity is always smooth except for boundaries, which means there is a big variation between small gradients (in smooth regions) and large gradients (near the boundaries). For out-of-focus images, the blurring effect brings the variation closer (i.e., smoothing the boundary and introducing fringes in the smooth region), which reduces the variance of gradients. As shown in Fig. S5(b), we plot the standard deviation of the gradients at regions around each bar with different propagation distance. There are peaks at 49.8 mm, 50 mm, and 50.2 mm for bar A, B, and C, respectively. Next, the in-focus object is recovered by back-propagating the field to the correct depth. Fig. S5(c) shows a 3D visualization of the result. This method is easy to implement for planar objects at various depths. But for objects with continuous depth variation, the region for calculating the metric needs to be chosen wisely.

**
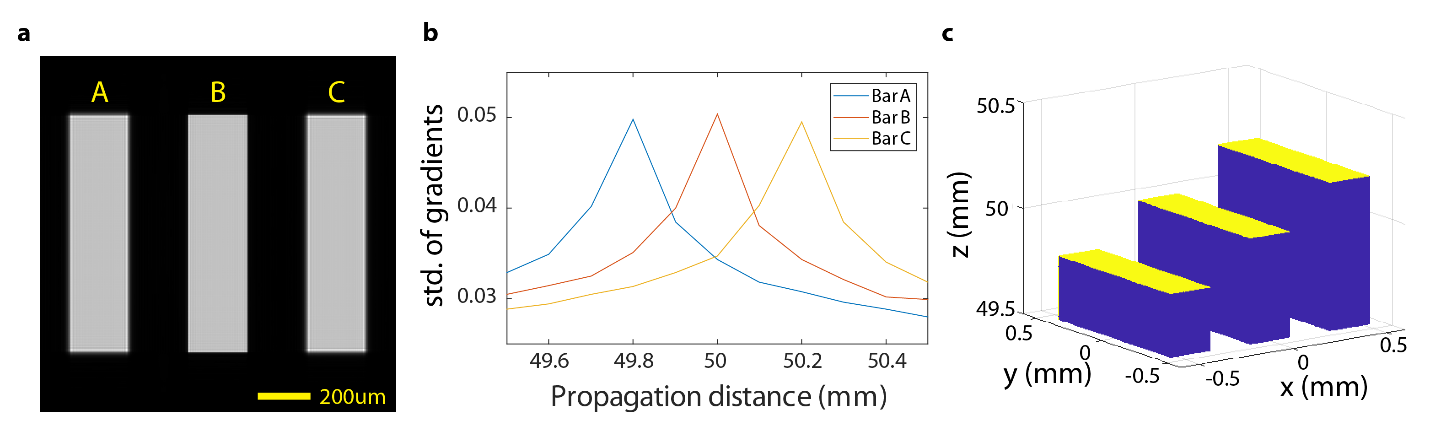
Figure S5: Depth estimation for planar objects at different depths based on the recovered amplitude.** Three planar bars (named as A, B, C) are located at 49.8 mm, 50 mm, and 50.2mm. **(a)** Recovered amplitude when choosing the propagation distance to be 50mm. Bar B is in-focus, and bar A and C is out-of-focus. **(b)** The standard deviation of gradients in A, B, C regions under different propagation distance. **(c)** 3D visualization of the objects.

Second, we can estimate depth based on the recovered phase. To do so, we need to change our current setup from transmissive mode to reflective mode, meaning that the incident light bounces back from the object instead of passing through it. Under the circumstances, the depth map is estimated by the phase distribution. Figure S6 gives one simulation example. It is a disk with quadratic phase distribution (Fig. S6a). Based on the phase distribution, we can calculate the 3D map of the object (Fig. S6b). Due to phase wrapping, the depth range is limited to half of the wavelength. Phase unwrapping^7^ is one way to extend the depth range.

**
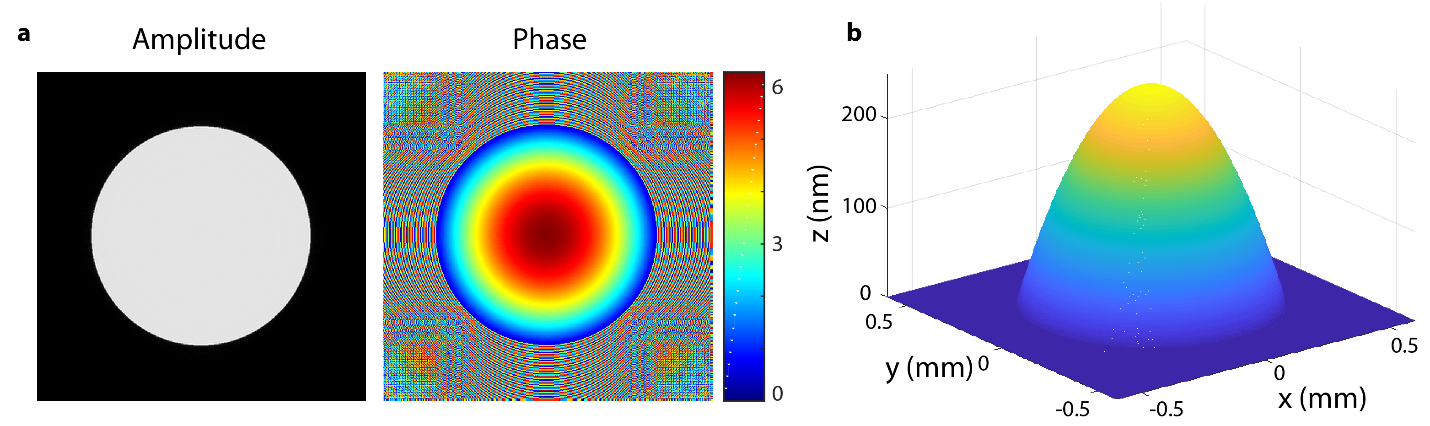
Figure S6: Depth estimation for a depth-varying object based on the recovered phase.** **(a)** The amplitude is a disk function and the phase is a quadratic function. **(b)** The estimated depth map of the object.

**Reference**

1. Goodman, J. Introduction to Fourier optics. (2008).

2. Vershynin, R. Introduction to the non-asymptotic analysis of random matrices. in *Compressed Sensing* (eds. Eldar, Y. C. & Kutyniok, G.) 210–268 (Cambridge University Press, 2009). doi:10.1017/CBO9780511794308.006

3. Candès, E. J., Li, X. & Soltanolkotabi, M. Phase retrieval from coded diffraction patterns. *Appl. Comput. Harmon. Anal.* **39,** 277–299 (2015).

4. Schmidt, J. D. *Numerical simulation of optical wave propagation with examples in MATLAB*. (SPIE, 2010).

5. Tian, L. & Waller, L. 3D intensity and phase imaging from light field measurements in an LED array microscope. *Optica* **2,** 104 (2015).

6. Kamilov, U. S. *et al.* Learning approach to optical tomography. *Optica* **2,** 517 (2015).

7. Bioucas-Dias, J. M. & Valadao, G. Phase Unwrapping via Graph Cuts. *IEEE Trans. Image Process.* **16,** 698–709 (2007).
